# Fabrication of Conducting Polyacrylate Resin Solution with Polyaniline Nanofiber and Graphene for Conductive 3D Printing Application

**DOI:** 10.3390/polym10091003

**Published:** 2018-09-08

**Authors:** Hoseong Han, Sunghun Cho

**Affiliations:** School of Chemical Engineering, Yeungnam University, Gyeongsan 38541, Korea; byecome123@gmail.com

**Keywords:** 3D printing, digital light processing, polyacrylate composite, polyaniline, graphene

## Abstract

Three-dimensional printing based on the digital light processing (DLP) method offers solution processability, fast printing time, and high-quality printing through selective light curing of photopolymers. This research relates to a method of dispersing polyaniline nanofibers (PANI NFs) and graphene sheets in a polyacrylate resin solution for optimizing the conductive solution suitable for DLP-type 3D printing. Dispersion and morphology of the samples with different filler contents were investigated by field emission scanning electron microscope (FE-SEM) and optical microscope (OM) analyses. The polyacrylate composite solution employing the PANI NFs and graphene was printed well with various shapes and sizes through the 3D printing of DLP technology. In addition, the electrical properties of the printed sculptures have been investigated using a 4-point probe measurement system. The printed sculpture containing the PANI NFs and graphene sheets exhibited electrical conductivity (4.00 × 10^−9^ S/cm) up to 10^7^ times higher than the pure polyacrylate (1.1 × 10^−16^ S/cm). This work suggests potential application of the PANI NF/graphene cofiller system for DLP-type 3D printing.

## 1. Introduction

Of the various technologies that can contribute to the Fourth Industrial Revolution, 3D printing is becoming a future-oriented manufacturing process because it can produce customized products of desired design, shape and size. In particular, 3D printing is considered to be an advanced polymer processing technology, since various polymers including polyurethane (PU), polyacrylate, polylactide (PLA), polystyrene (PS), acrylonitrile butadiene styrene (ABS) resin, and polyvinyl chloride (PVC) are used as raw materials [1,2].

Methods for 3D printing are divided into several categories, such as material extrusion, digital light processing (DLP), stereolithography (SLA), powder bed fusion, material jetting, binder jetting, powder jet fusion, and so forth. Material extrusion is a 3D printing process in which molten polymers are selectively dispensed through the nozzle [1]. Medical 3D printing, including artificial bones, artificial organs, and medical devices, is a representative example of the extrusion-type 3D printing method [2,3]. However, 3D printing based on material extrusion usually suffers from low printing quality and long processing time. The DLP method enables selective light curing of photopolymer solutions by generating ultraviolet (UV) light from the projector [4,5,6]. Therefore, 3D printing using DLP provides shorter printing time and better printing quality compared to material extrusion. Furthermore, the DLP method is advantageous for realizing an improved dispersion in the polymer compounding, thereby maximizing the synergistic effect between the polymer matrix and the fillers [5,6].

If the printed material is electrically conductive, it will be possible to expand the application range of polymers for 3D printing and additive manufacturing. Various efforts have been made to fabricate 3D conductive structures using the DLP method [5,6]. In addition, Wicker et al. have reported the combination of vat polymerization with direct writing technology for printing a 3D conductive circuit [7]. Despite these technological advances, the types of conductive materials that can be used for DLP-type 3D printing are still limited. In order to overcome these limitations, it is essential to diversify the conducting additives for DLP-type 3D printing that can be highly dispersible with the polymer resin.

Conducting polymers, such as polyaniline (PANI), poly(3,4-ethylenedioxythiophene) (PEDOT), polypyrrole (PPy), and polythiophene (PT), have a conjugated system structure and are therefore defined as polymeric materials capable of exhibiting electrical conductivity after appropriate doping [8,9]. These conducting polymers are also characterized by the advantages of conventional polymers such as processability, light weight, and low cost. In particular, PANI exhibits the most various oxidation and reduction behaviors, and PANI can achieve almost the same conductivity even though it is about one-hundredth of the price of PEDOT [10,11,12,13]. In addition, since PANI can be obtained by nanoscale fabrication, including nanofibers (NFs), nanorods (NRs), nanoparticles (NPs), and nanotubes (NTs), a denser conductive path can be formed inside the polymer resin because of improved surface area and electrochemical activity [10,11,12,13]. For these reasons, PANI and aniline tetramer are also considered to be among the most attractive candidates for conducting fillers in 3D printing [14,15,16]. However, polymeric materials such as PANI are vulnerable to heat and light, so they are highly likely to lose their inherent electrical properties during 3D printing. It is known that the limitations of PANI can be largely solved when the graphene sheet is used as a filler [12,13,16]. Graphene has an advantage of being able to improve the reliability and stability of the resulting products because it provides excellent electrical conductivity, thermal stability, and mechanical stability at the same time [16,17]. Considering the above facts, it is necessary to study the conductive 3D printing based on the DLP method using a polymer resin solution in which PANI NF and graphene sheets are dispersed at the same time.

In this work, PANI NFs and graphene sheets were introduced into a polyacrylate resin having excellent processability and applicability, and DLP-type 3D printing using the conductive polyacrylate solution was discussed. Field emission scanning electron microscope (FE-SEM) and optical microscope (OM) images were used to investigate the presence and dispersion of conductive fillers. The role of PANI NF/graphene cofiller dispersed in the polyacrylate resin was confirmed by measuring the surface resistance and electrical conductivity of 3D printed sculptures. The printed sculpture containing the optimal amount of PANI NF/graphene cofiller exhibited high printability and improved electrical properties, suggesting that the PANI NF/graphene cofiller system can provide a unique approach to design DLP-type 3D printing.

## 2. Materials and Methods

Aniline (99%) and ammonium persulfate (APS, 98%) were obtained from Sigma-Aldrich (St. Louis, MO, USA). Graphene paste was obtained from MExplorer Co., Ltd. (Ansan, Korea). The graphene sheet has a density of 25 mg/mL, and the average thickness and lateral size of the graphene sheet are approximately <5 nm and 2–3 μm, respectively. The polyacrylate resin solution (Carima Acryl, 3DK83I) was obtained from Carima (Seoul, Korea). Hydrochloric acid (HCl, 35–37%), ethanol (95%), and acetone (99%) were purchased from Daejung Chemical & Metals Co., Ltd. (Siheung, Korea). The PANI NFs used in this experiment were prepared by a chemical oxidation polymerization method which was optimized from our previous work [10]. 1.1 × 10^−2^ mol of aniline was added to 40 mL of 1M aqueous HCl solution and stirred for about 30 min. After that, 5.3 × 10^−3^ mol of APS was added and reacted at room temperature for 3 h to obtain emerald-colored PANI precipitates. The prepared PANI precipitates were washed with water, ethanol and acetone solvent.

Preparative conditions of polyacrylate solutions are shown in Table 1. The PANI precipitate (5 wt % with respect to polyacrylate solution) prepared through the above processes was inserted and dispersed in a polyacrylate resin solution. In addition, graphene sheets were added to the resin solution at different weight fractions of 0.3, 0.6, and 1.2 wt %, respectively. The dispersion treatment of the conductive fillers was completed through magnetic stirring for 5 h at a stirring speed of 600 rpm and sonication treatment for 3 h. The sonication treatments of the samples were carried out using an ultrasonic bath (CPX2800H-E, Branson Ultrasonics Co., Danbury, CT, USA) with 110 W power and 40 kHz frequency. To maintain the dispersion temperature at 25 ± 2 °C, we replaced the cold water in the ultrasonic bath every 20 min.

The 3D printer used in this experiment was a DLP-type printing system (IM-96, Carima, Seoul, Korea). The resin solution containing different amounts of conductive fillers was 3D printed and evaluated for printability. The maximum printable concentration of PANI NF was 5 wt % and no lamination occurred when the content of PANI NF exceeded 5 wt %. In the case of graphene, 3D printing could be achieved at a content of less than 2 wt % by weight. Based on the microscope images and the results of printability, the weight ratios of PANI NF to graphene controlled in this work were 1.0:0.06 (PG1), 1.0:0.12 (PG2), and 1.0:0.25 (PG3), respectively.

Images of the fillers and 3D printed samples were obtained with a FE-SEM (S-4800, HITACHI, LTD, Hitachi, Japan) and OM (Nikon Lv100 microscope, Nikon, Japan). Electrical properties of the 3D printed samples were conducted using a 4-point probe conductivity meter (Mode Systems Co., Hanam, Korea) equipped with a current source meter (Keithley 2400, Keithley Co., Cleveland, OH, USA). The electric conductivity (σ) measurement formula by the 4 point probe conductivity method is defined as σ (S/cm) = 1/*ρ* = (ln2/π*t*)(I/V), where *ρ* is the static resistivity, *R* is the surface resistance, and *t* is the thickness of the sample [8].

## 3. Results

**Figure 1:**
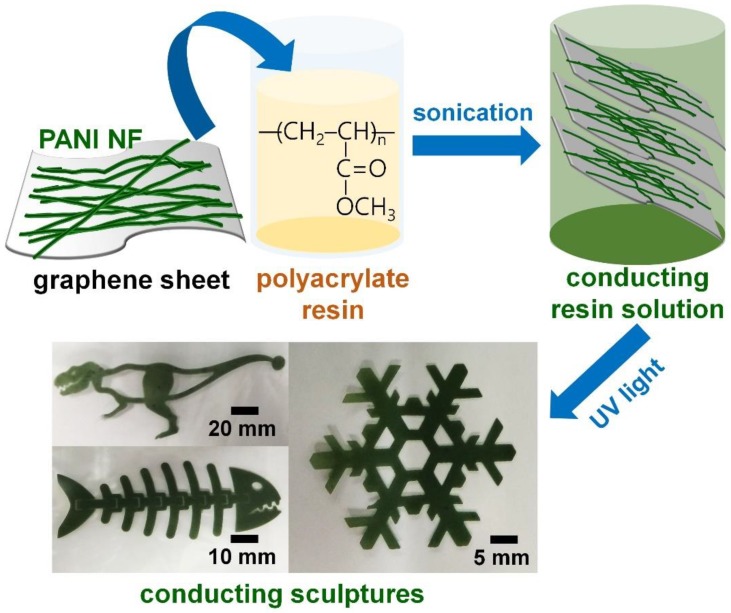
Overall process of conductive 3D printing using polyacrylate resin solution employing PANI NF (polyaniline nanofibers) and graphene sheet.

Figure 1 summarizes the preparation of the conductive polyacrylate solution and the 3D printing process. PANI NFs and graphene sheets were dispersed in a polyacrylate resin solution containing a photocrosslinking agent through mechanical stirring and ultrasonic treatment. The conductive fillers form a conductive passage in the polyacrylate resin, thereby enabling the electrically conductive sculptures to be realized even after the 3D printing was completed. PANI NFs have a high aspect ratio, which reduces intermaterial resistance and contact resistance of the composite [9,10,12]. In addition, the graphene sheet also serves as a support to prevent the PANI polymer from swelling or pyrolysis [12,13,14]. The polyacrylate resin used in this experiment will serve as a matrix for maintaining the desired shape and size after 3D printing and the dispersion medium of the conductive filler. When 3D printing is performed using UV light having a wavelength of about 300 nm, conductive sculptures having various shapes and sizes can be easily manufactured by photocrosslinking between polyacrylate chains.

**Figure 2:**
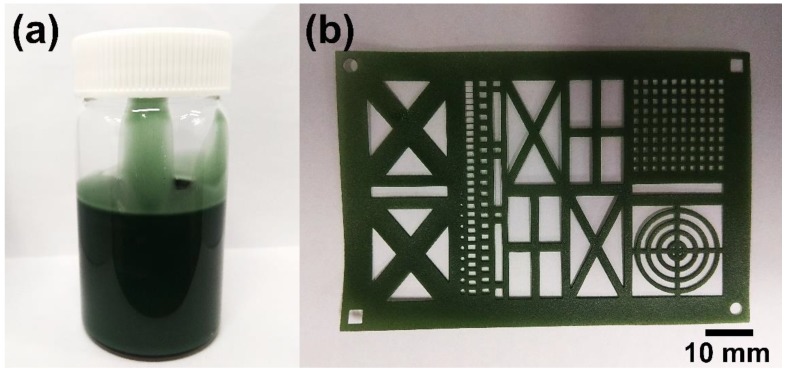
Digital images of (**a**) a conducting resin solution and (**b**) a 3D printed sculpture.

Figure 2 shows the digital images of the conductive polyacrylate solutions and sculptures actually made. The dispersed form of the conductive fillers was emerald colored and remained stable even after 30 days without phase separation (Figure 2a). This means that the dispersion process of the conductive fillers used in the present experiment was appropriately performed. As a result of performing 3D printing using the above solutions, it has been confirmed that the conductive sculptures can be manufactured with various sizes and complex patterns (Figure 2b).

**Figure 3:**
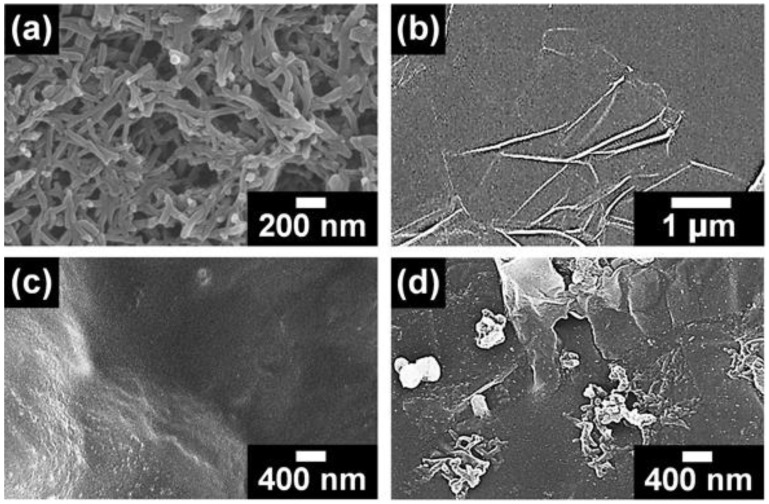
Field emission scanning electron microscope (FE-SEM) images of (**a**) PANI NF, (**b**) graphene, (**c**) pristine polyacrylate and (**d**) polyacrylate/PANI NF/graphene composite.

Figure 3 shows FE-SEM images of PANI, graphene, pristine polyacrylate, and polyacrylate/PANI NF/graphene composite. Diameters and lengths of the PANI NFs were 40–60 nm and 0.6–1.5 μm, respectively (Figure 3a). The sizes of graphene sheets ranged from 2 to 5 μm (Figure 3b). No filler was found in the FE-SEM image of the pristine polyacrylate resin, indicating that the resin acted as a matrix of PANI NF and graphene (Figure 3c). These PANI NFs and graphene were dispersed in the polyacrylate resin at different filler contents (Figures S1–S3, see Supplementary Materials). In Figure S1, it was found that the number of PANI NFs present in the same area increased as the filler content increased. No remarkable aggregation of PANI NF was found until the PANI NF content reached 5 wt %, while the sample containing 10 wt % of PANI NFs exhibited remarkable aggregation (Figure S1a–d). The results of the OM images were also consistent with the tendency shown in the FE-SEM images (Figure S3). These results indicate that the aggregation of PANI NFs disturb the lamination of polyacrylate resin; therefore, the optimum amount of PANI in the polyacrylate resin has been fixed at 5 wt %. FE-SEM images of the samples containing different amounts of graphene are shown in Figure S2a–d. Excessive aggregation of graphene sheets was observed at the graphene content of 2.5 wt %, which is associated with the van der Waals interactions between each graphene sheet (Figure S2c) [12,16,17]. The size of graphene clusters observed in OM images also increased significantly at a graphene content of 2.5 wt % (Figure S3). Therefore, the maximum amount of graphene added in the 3D printable resin was adjusted to 1.2 wt %. Based on the results of FE-SEM and OM images, 5 wt % of PANI NF and 1.2 wt % of graphene were introduced into the polyacrylate resin (Figure 3d). Compared with Figure 3c, it was evident that both the PANI and graphene were embedded in the polyacrylate resin. In Figure 3d, some of the PANI NFs were observed to be shorter than the initial length of the PANI NFs shown in Figure 3a. This phenomenon may be due to several reasons: (1) According to previous reports, polymeric binders play a role in reducing the size and length of nanomaterials [18,19]. (2) It is known that the continuous ultrasound can destroy polymer chains, which results in the destruction of the nanostructures [20]. Therefore, it is considered that both factors affect the resultant morphology of PANI NF. Judging from these results, it was confirmed that the PANI NFs and graphene were successfully introduced into the polyacrylate resin at appropriate filler contents.

**Figure 4:**
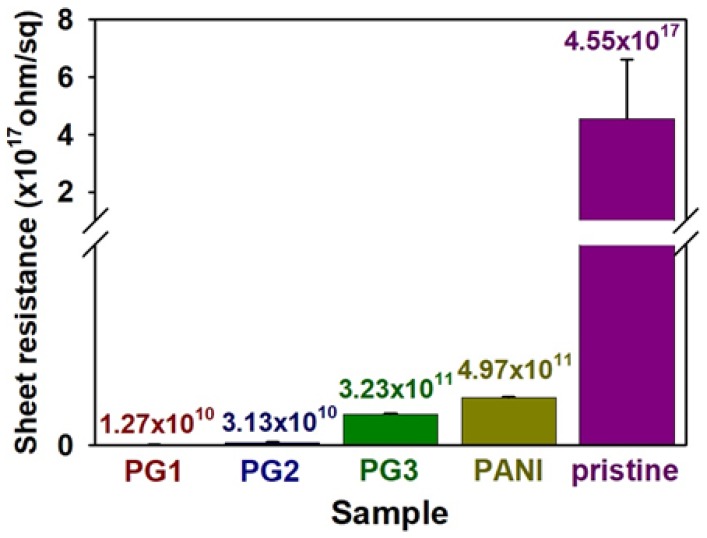
Sheet resistance of pristine polyacrylate and composites employing conductive fillers: PANI (5 wt % PANI NFs), PG1 (5 wt % PANI and 1.2 wt % graphene), PG2 (5 wt % PANI and 0.6 wt % graphene), PG3 (5 wt % PANI and 0.3 wt % graphene).

Figure 4 shows the sheet resistance of the polyacrylate composites employing the PANI NF and graphene sheet. The average sheet resistance of the polyacrylate added with 5 wt % of PANI NFs was about 4.97 × 10^11^ Ω/sq, which was about 10^6^ times lower than that of the pure polyacrylonitrile resin (4.55 × 10^17^ Ω/sq). Nevertheless, the fact that the sheet resistance value is higher than 10^10^ Ω/sq is considered to be due to the decomposition of PANI NFs during the photocrosslinking reaction, and the deterioration of electrical properties due to the fact that most of the PANI NFs are surrounded by the resin. In order to solve the problem, graphene sheets of 0.3, 0.6, and 1.2 wt % were introduced into the polyacrylate resin containing PANI NFs and then 3D printing was performed. The sheet resistance of the sample became lower when the amount of graphene sheet was increased: pristine polyacrylate (4.55 × 10^17^ Ω/sq) < 0.3 wt % (3.23 × 10^11^ Ω/sq) < 0.6 wt % (3.13 × 10^10^ Ω/sq) < 1.2 wt % (1.27 × 10^10^ Ω/sq). The result indicates that when the content of the graphene sheet exceeds 0.6 wt %, the restacking and agglomeration between the sheets are intensified, and the reduction of resistance thereby becomes stagnant. Therefore, in this experiment, it was judged that optimum electrical properties were realized at an additional amount of 1.2 wt % of graphene [12,16,17].

**Figure 5:**
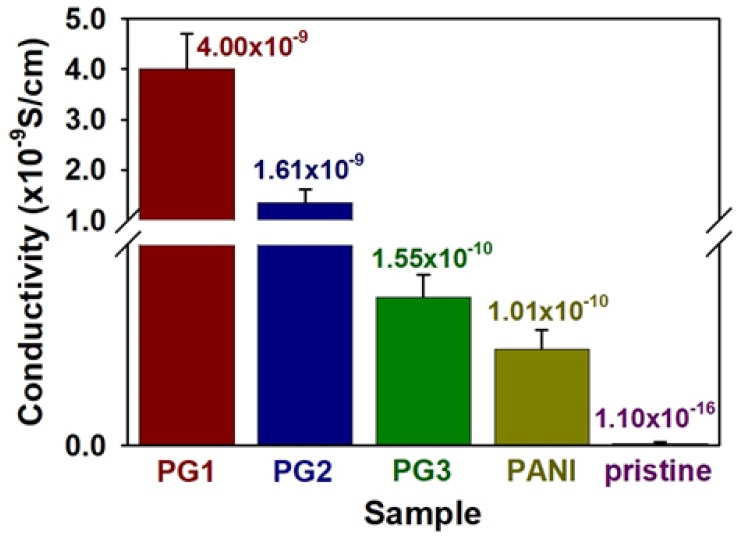
Electrical conductivity of pristine polyacrylate and composites employing conductive fillers: PANI (5 wt % PANI NFs), PG1 (5 wt % PANI and 1.2 wt % graphene), PG2 (5 wt % PANI and 0.6 wt % graphene), PG3 (5 wt % PANI and 0.3 wt % graphene).

To achieve the practical measurements of the polyacrylate composites, electrical conductivity of the samples was calculated according to the 4-point probe conductivity method (Figure 5) [8]. Electrical conductivity of the pristine polyacrylate was measured to be 1.1 × 10^−16^ S/cm, indicating that the pristine resin is an almost insulating material. After adding 5 wt % of PANI NFs to polyacrylate resin, the measured conductivity value was 1.01 × 10^−10^ S/cm. The electrical conductivity of the composites employing the graphene sheets of 0.3, 0.6, and 1.2 wt % was 1.55 × 10^−10^, 1.61 × 10^−9^, and 4.00 × 10^−9^ S/cm, respectively. The results reconfirm that appropriate amounts of the graphene sheet and PANI NF provide effective conductive pathways within the polyacrylate matrix [12,16,17].

## 4. Conclusions

In this study, DLP-type 3D printing of polyacrylate composites employing PANI NF and graphene dispersion was investigated. The presences of PANI NFs and graphene were clarified using FE-SEM and OM images, and the optimal contents of PANI and graphene were 5 wt % and 1.2 wt %, respectively. The prepared NFs were highly dispersible with the polyacrylate resin, resulting in a high electrical conductivity value of about 10^6^ times. In addition, when a proper amount (1.2 wt % with respect to polyacrylate resin solution) of graphene sheet was introduced, an additional electrical conductivity improvement of about 40 times occurred. The results mean that even after 3D printing is complete, the conductive fillers provide conductive regions within the polyacrylate resin. The results also have shown that the conductive 3D printing solution prepared by our work can be reprocessed as conductive sculptures with various designs and sizes. The sheet resistance and electrical conductivity values obtained from the results are enough to be used as conventional conductive resins for antistatic property. It is expected that the progress of DLP-type 3D printing technology using a PANI NF/graphene cofiller system and its expansion of application fields, such as electronic and biomedical devices, will be further accelerated.

## Figures and Tables

**Table 1:** Preparative conditions of polyacrylate resin solutions.

Sample	Polyacrylate (g)	PANI NFs (g)	Graphene (g)
pristine	20.0	-	-
PANI	19.0	1.0 ^1^	-
PG1	18.94	1.0 ^1^	0.06 ^2^
PG2	18.88	1.0 ^1^	0.12 ^3^
PG3	18.75	1.0 ^1^	0.25 ^4^

^1^ 5.0 wt % with respect to polyacrylate solution. ^2^ 0.3 wt % with respect to polyacrylate solution. ^3^ 0.6 wt % with respect to polyacrylate solution. ^4^ 1.2 wt % with respect to polyacrylate solution.

## Supplementary Materials

The following are available online at http://www.mdpi.com/2073-4360/10/9/1003/s1, Figure S1: FE-SEM images of polyacrylate composites containing different PANI NF content, Figure S2: FE-SEM images of polyacrylate composites containing different graphene content, Figure S3: OM images of polyacrylate composites containing different contents of PANI NF and graphene.

## References

[1] Ligon S.C., Liska R., Stampfl J., Gurr M., Mülhaupt R. (2017). Polymers for 3D Printing and Customized Additive Manufacturing. Chem. Rev..

[2] Stansbury J.W., Idacavage M.J. (2016). 3D printing with polymers: Challenges among expanding options and opportunities. Dent. Mater..

[3] Kankala R.K., Xu X.M., Liu C.G., Chen A.Z., Wang S.B. (2018). 3D-Printing of Microfibrous Porous Scaffolds Based on Hybrid Approaches for Bone Tissue Engineering. Polymers.

[4] Patel D.K., Sakhaei A.H., Layani M., Zhang B., Ge Q., Magdassi S. (2017). Highly Stretchable and UV Curable Elastomers for Digital Light Processing Based 3D Printing. Adv. Mater..

[5] Mu Q., Wang L., Dunn C.K., Kuang X., Duan F., Zhang Z., Qi H.J., Wang T. (2017). Digital light processing 3D printing of conductive complex structures. Addit. Manuf..

[6] Cullen A.T., Price A.D. (2018). Digital light processing for the fabrication of 3D intrinsically conductive polymer structures. Synth. Met..

[7] Espalin D., Muse D.W., MacDonald E., Wicker R.B. (2014). 3D Printing multifunctionality: Structures with electronics. Int. J. Adv. Manuf. Technol..

[8] Skotheim T.A., Elsenbaumer R.L., Shacklette L.W. (2007). Handbook of Conducting Polymers.

[9] Jang J. (2006). Conducting polymer nanomaterials and their applications. Adv. Polym. Sci..

[10] Cho S., Kwon O.S., You S.A., Jang J. (2013). Shape-controlled polyaniline chemiresistors for high-performance DMMP sensors: Effect of morphologies and charge-transport properties. J. Mater. Chem. A.

[11] Pang S., Chen W., Yang Z., Liu Z., Fan X., Fang D. (2017). Facile Synthesis of Polyaniline Nanotubes with Square Capillary Using Urea as Template. Polymers.

[12] Cho S., Kim M., Lee J.S., Jang J. (2015). Polypropylene/Polyaniline Nanofiber/Reduced Graphene Oxide Nanocomposite with Enhanced Electrical, Dielectric, and Ferroelectric Properties for a High Energy Density Capacitor. ACS Appl. Mater. Interfaces.

[13] Shahabuddin S., Sarih N.M., Kamboh M.A., Nodeh H.R., Mohamad S. (2016). Synthesis of Polyaniline-Coated Graphene Oxide@SrTiO_3_ Nanocube Nanocomposites for Enhanced Removal of Carcinogenic Dyes from Aqueous Solution. Polymers.

[14] Lu X., Zhao T., Ji X., Hu J., Li T., Lin X., Huang W. (2018). 3D printing well organized porous iron-nickel/polyaniline nanocages multiscale supercapacitor. J. Alloys Compd..

[15] Dong S.L., Han L., Du C.X., Wang X.Y., Li L.H., Wei Y. (2017). 3D Printing of Aniline Tetramer-Grafted-Polyethylenimine and Pluronic F127 Composites for Electroactive Scaffolds. Macromol. Rapid Commun..

[16] Wang Z., Zhang Q., Long S., Luo Y., Yu P., Tan Z., Bai J., Qu B., Yang Y., Shi J. (2018). Three-Dimensional Printing of Polyaniline/Reduced Graphene Oxide Composite for High-Performance Planar Supercapacitor. ACS Appl. Mater. Interfaces.

[17] Gaska K., Xu X., Gubanski S., Kádár R. (2017). Electrical, Mechanical, and Thermal Properties of LDPE Graphene Nanoplatelets Composites Produced by Means of Melt Extrusion Process. Polymers.

[18] Syed J.A., Tang S., Lu H., Meng X. (2015). Water-Soluble Polyaniline–Polyacrylic Acid Composites as Efficient Corrosion Inhibitors for 316SS. Ind. Eng. Chem. Res..

[19] Park H., Kim T., Huh J., Kang M., Lee J., Yoon H. (2012). Anisotropic Growth Control of Polyaniline Nanostructures and Their Morphology-Dependent Electrochemical Characteristics. ACS Nano.

[20] Zhang K., Park B.J., Fang F.F., Choi H.J. (2009). Sonochemical Preparation of Polymer Nanocomposites. Molecules.

