# Supplementary material for: Fabrication of Conducting Polyacrylate Resin Solution with Polyaniline Nanofiber and Graphene for Conductive 3D Printing Application
Source: Polymers (Basel). 2018 Sep 8;10(9):1003. doi: 10.3390/polym10091003 (PMC6403923; doi:10.3390/polym10091003)
Supplement: Supplementary file 1 [file polymers-10-01003-s001.pdf]

Supplementary Materials for:

Fabrication of Conducting Polyacrylate Resin Solution with Polyaniline Nanofiber and Graphene for Conductive 3D Printing Application

Hoseong Han[1] and Sunghun Cho[1,*]

[1] School of Chemical Engineering, Yeungnam University, Gyeongsan 38541, Republic of Korea.

*E-mail: shcho83@ynu.ac.kr
Tel.: +82-53-810-535

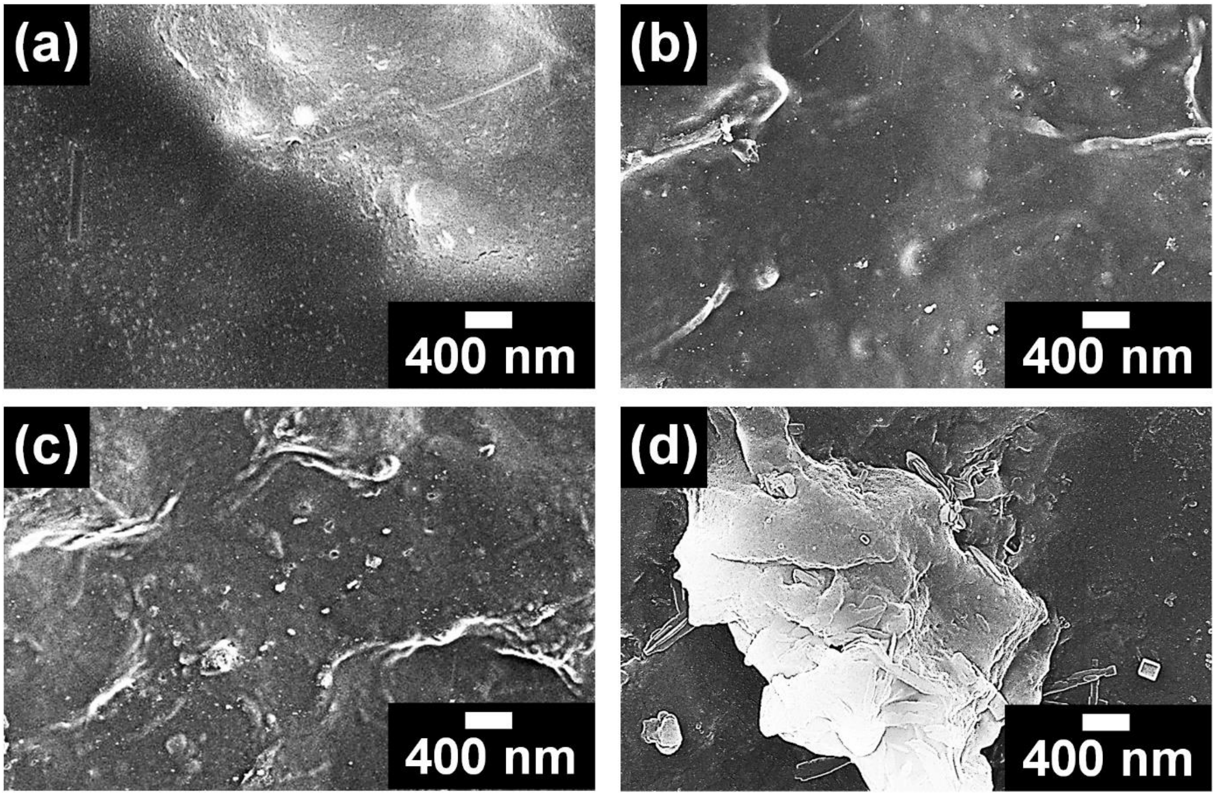

Figure S1. FE-SEM images of polyacrylate composites containing different PANI NF content: (a) 1 wt %, (b) 2 wt %, (c) 5 wt %, and (d) 10 wt %.

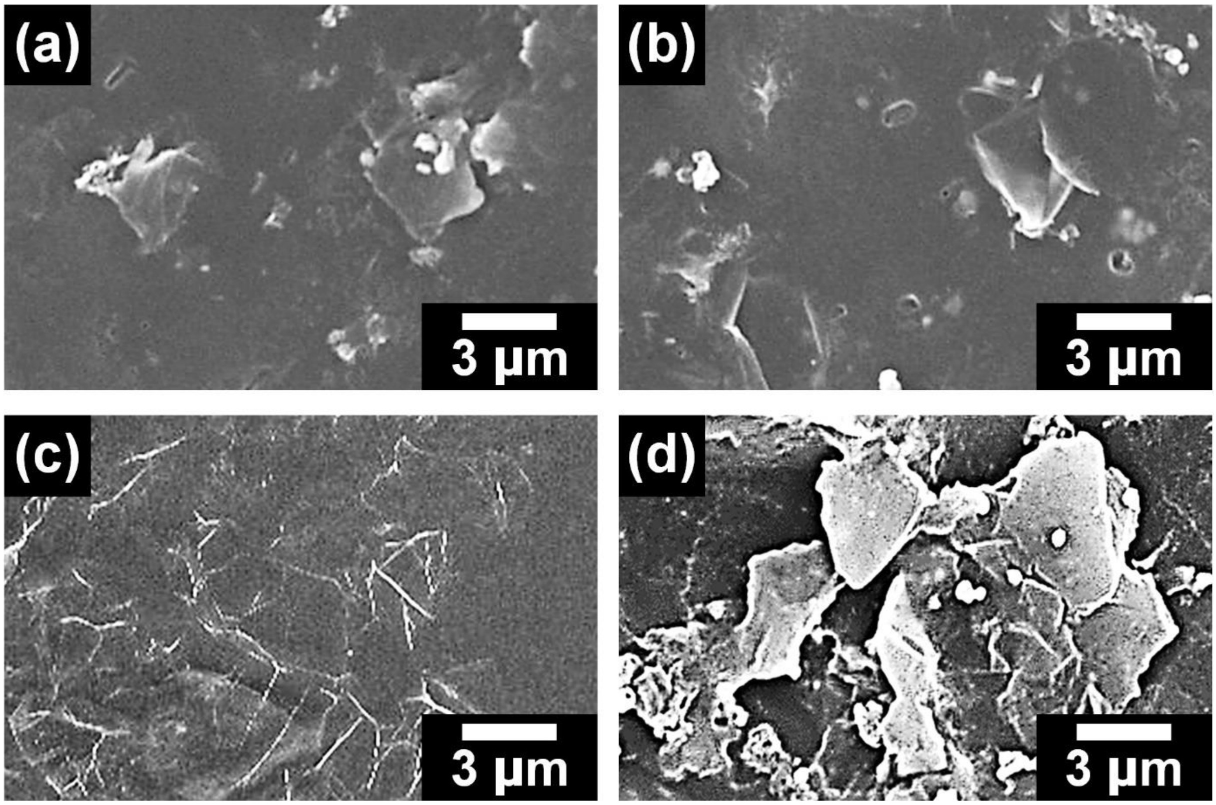

Figure S2. FE-SEM images of polyacrylate composites containing different PANI NF content: (a) 0.3 wt %, (b) 0.6 wt %, (c) 1.2 wt %, and (d) 2.5 wt %.

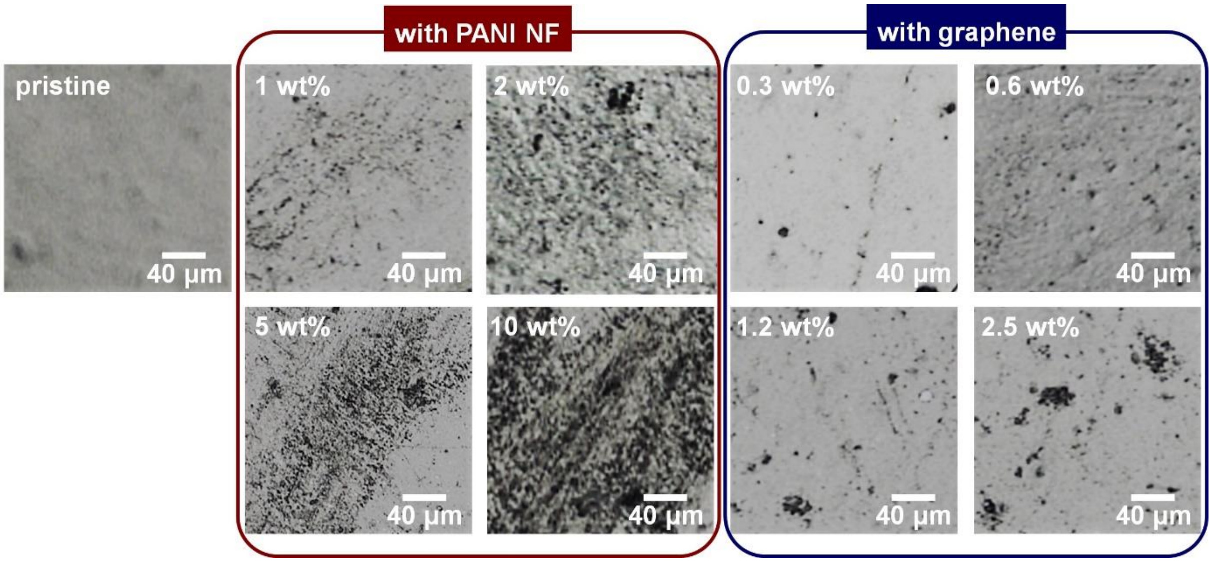

Figure S3. OM images of polyacrylate composites containing different contents of PANI NF and graphene.
